# Enhanced microglial pro‐inflammatory response to lipopolysaccharide correlates with brain infiltration and blood–brain barrier dysregulation in a mouse model of telomere shortening

**DOI:** 10.1111/acel.12370

**Published:** 2015-08-03

**Authors:** Divya D. A. Raj, Jill Moser, Susanne M. A. van der Pol, Ronald P. van Os, Inge R. Holtman, Nieske Brouwer, Hisko Oeseburg, Wandert Schaafsma, Evelyn M. Wesseling, Wilfred den Dunnen, Knut P. H. Biber, Helga E. de Vries, Bart J. L. Eggen, Hendrikus W. G. M. Boddeke

**Affiliations:** ^1^ Section Medical PhysiologyDepartment of NeuroscienceUniversity of GroningenUniversity Medical Center GroningenGroningen9713 AVThe Netherlands; ^2^ Department of Critical CareUniversity of GroningenUniversity Medical Center GroningenGroningen9713 AVThe Netherlands; ^3^ Department of Pathology and Medical BiologyUniversity of GroningenUniversity Medical Center GroningenGroningen9713 AVThe Netherlands; ^4^ Blood‐Brain Barrier Research GroupDepartment of Molecular Cell Biology and ImmunologyNeuroscience Campus AmsterdamVU University Medical CentrePO Box 70571007 MBAmsterdamThe Netherlands; ^5^ Department of Cell BiologyEuropean Research Institute on the Biology of AgingUniversity of GroningenUniversity Medical CenterGroningen9713 AVThe Netherlands; ^6^ Department of CardiologyUniversity Medical Center GroningenGroningen9713 AVThe Netherlands; ^7^ Department of Psychiatry and PsychotherapyUniversity Medical CenterFreiburg79104Germany

**Keywords:** aging, blood–brain barrier, microglia, neuroimmune response, priming, telomere, telomerase

## Abstract

Microglia are a proliferative population of resident brain macrophages that under physiological conditions self‐renew independent of hematopoiesis. Microglia are innate immune cells actively surveying the brain and are the earliest responders to injury. During aging, microglia elicit an enhanced innate immune response also referred to as ‘priming’. To date, it remains unknown whether telomere shortening affects the proliferative capacity and induces priming of microglia. We addressed this issue using early (first‐generation G1 mTerc^−/−^)‐ and late‐generation (third‐generation G3 and G4 mTerc^−/−^) telomerase‐deficient mice, which carry a homozygous deletion for the telomerase RNA component gene (*mTerc*). Late‐generation mTerc^−/−^ microglia show telomere shortening and decreased proliferation efficiency. Under physiological conditions, gene expression and functionality of G3 mTerc^−/−^ microglia are comparable with microglia derived from G1 mTerc^−/−^ mice despite changes in morphology. However, after intraperitoneal injection of bacterial lipopolysaccharide (LPS), G3 mTerc^−/−^ microglia mice show an enhanced pro‐inflammatory response. Nevertheless, this enhanced inflammatory response was not accompanied by an increased expression of genes known to be associated with age‐associated microglia priming. The increased inflammatory response in microglia correlates closely with increased peripheral inflammation, a loss of blood–brain barrier integrity, and infiltration of immune cells in the brain parenchyma in this mouse model of telomere shortening.

## Introduction

Microglia, the resident macrophages of the brain, are derived from early yolk sac macrophage progenitors (Ginhoux *et al*., 2010; Kierdorf *et al*., 2013). Under physiological conditions, there is negligible infiltration of peripheral monocytes to the brain parenchyma and the microglia population is likely sustained by self‐renewal (Ajami *et al*., 2007). Although, there have been previous studies aiming to understand the cycling rate of microglia (Lawson *et al*., 1992), the contribution of microglia proliferation to sustain the population in the brain during organismal lifespan remains unknown.

The length of telomeres in somatic cells shortens progressively with successive cell divisions (Hayflick, 1965), and telomere erosion has been shown to correlate with proliferative activity of cellular populations over lifespan (Allsopp *et al*., 1995). The intricate association between telomere shortening, cellular senescence, and organismal aging has been previously acknowledged (Campisi & d'Adda di Fagagna, 2007). As microglia represent a proliferation‐competent cell population, it is reasonable to hypothesize that self‐renewal and subsequent telomere shortening can alter the proliferative capacity and functionality of microglia. Indeed, microglia telomere shortening has been associated with increasing age and dementia (Flanary *et al*., 2007). The proliferative capacity of microglia has shown to be linked to its innate immune response (Shankaran *et al*., 2007) and seems to be a critical component in the evolution of chronic neurodegeneration (Gomez‐Nicola *et al*., 2013). In this study, we investigated whether telomere shortening could potentially alter the inflammatory reactivity of microglia in the brain.

Microglia in the aged brain have been shown to be primed for activation, a state of exaggerated inflammatory reactivity and persistent neuroinflammation. Microglia priming is considered an important confounding factor in age‐associated neurodegenerative diseases (Perry & Holmes, 2014). Dystrophic microglia, characterized by loss of structural integrity, the presence of spheroid inclusions, and fragmented cellular processes, have been reported in the aged human brain (Streit *et al*., 2004). Similar dystrophic microglia have been reported in rodent mouse models of accelerated aging and neurodegeneration (Hasegawa‐Ishii *et al*., 2011). Microglia from aged mice and Alzheimer's mouse models were shown to phagocytose and degrade less amyloid beta peptide (Aβ), thereby contributing to the disease process (Hickman *et al*., 2008; Njie *et al*., 2012). As dystrophy in microglia is restricted to aged and neurodegenerative brain tissues, it has been proposed to be the consequence of age‐associated telomere shortening and replicative senescence in microglia (Flanary *et al*., 2007).

It is presently unclear whether telomere shortening *in vivo* has a direct impact on microglia functionality. Understanding the impact of telomere shortening on microglia functionality is important and relevant to the aged brain. In this study, we show that in spite of telomere shortening, the basal physiological properties of microglia are largely unchanged. The increased microglial response to an inflammatory stimulus in G3 mTerc^−/−^ mice is likely mediated by a decline of blood–brain barrier integrity and increased immune cell infiltration upon telomere shortening.

## Materials and methods

### Animals

The telomerase knockout mice carry a homozygous deletion for the telomerase RNA component (*mTerc*) gene, which leads to complete loss of *Terc* expression and telomerase activity (reported in Herrera *et al*., 1999 in a C57/BL6 background). The mTerc^−/−^ breeding pairs were obtained from Jax mice (Stock number: 004132). All mice were maintained on the C57BL/6J background. A loss of more than 10% body weight within a week was decided as ethical endpoint. In the microarray experiment, 4‐month‐old G1 mTerc^−/−^ or G4 mTerc^−/−^ mice were used. B6.Cg‐Tg(CAG‐DsRed* MST)1Nagy/J (dsRed) transgenic mice originally purchased from the Jackson Laboratory, California, USA and bred in‐house. Young (3 months) and aged (24 months) C57BL/6J mice were purchased from Harlan, Boxmeer, the Netherlands. The aging animals were group‐housed under standard housing conditions with a 12‐h light–dark alternating cycles and standard chow diet *ad libitum* (ab diets; Cat. No. 2103). The mTerc^−/−^ mouse lines were maintained on 19% protein extruded rodent diet (T2919.10; Harlan Laboratories). All experiments were approved by the animal experimentation committee of the UMCG.

### Acute isolation of microglia and phenotyping using flow cytometry

The procedure is as described in Raj *et al*. (2014). The list of antibodies used for phenotyping is summarized in Table S1.

### RNA isolation and quantitative PCR analysis

The procedure is as described in Raj *et al*. (2014). The details of primers used are listed in Table S2.

## Results

### Genetic deletion of the *mTerc* gene results in telomere shortening and reduced proliferative capacity in microglia

**Figure 1:**
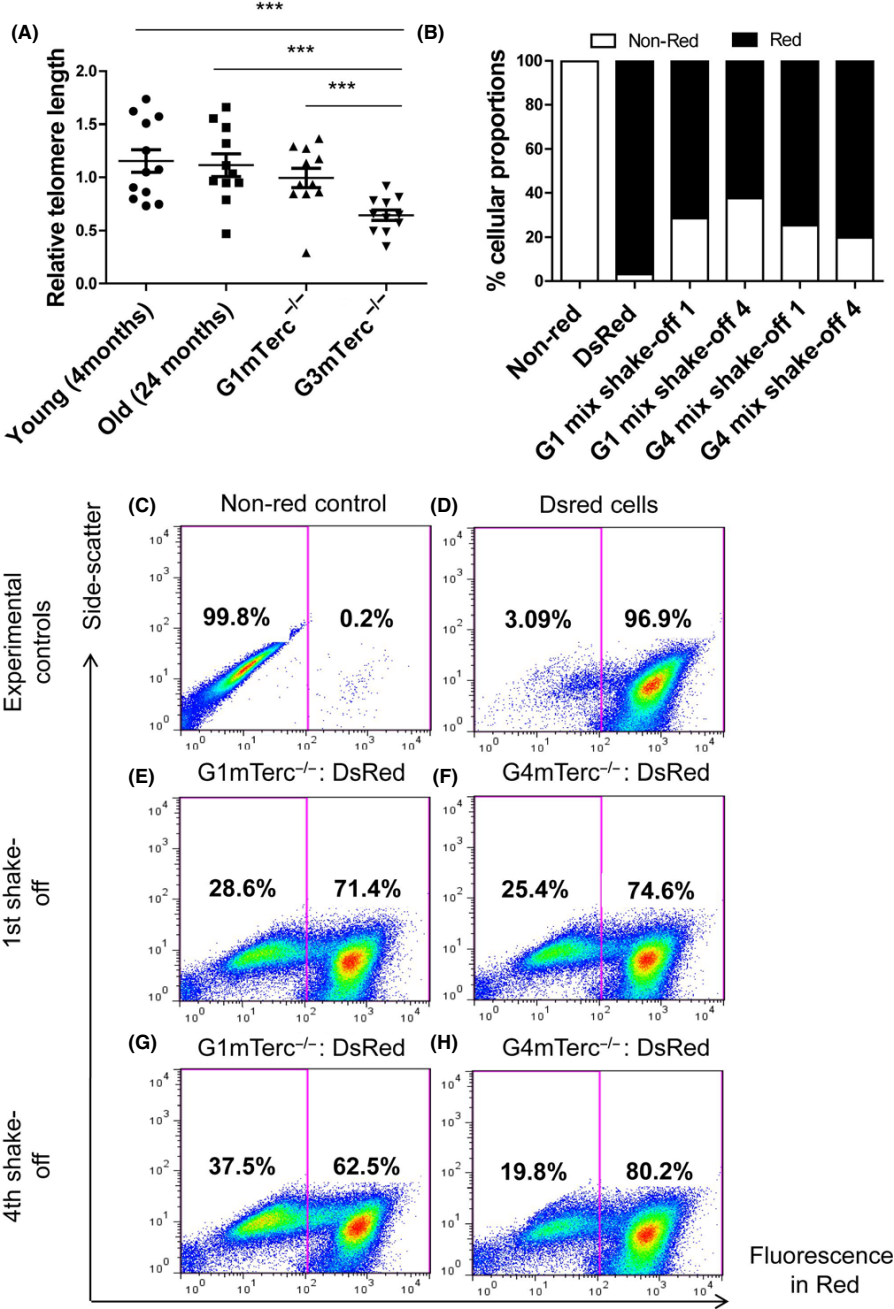
Telomerase ablation results in telomere shortening and a proliferative deficit in microglia. (A) Relative telomere length measurement in isolated microglia from young (4 months; *n* = 12) and old (24 months; *n* = 11) mice shows no significant difference, while isolated microglia from G1(*n* = 11) and G3 mTerc^−/−^ (*n* = 11) mice (6 months) have significantly shorter telomeres in G3 mTerc^−/−^ microglia. Asterisks * indicate comparisons for which *P*‐value was significant according to one‐way ANOVA *P < 0.05, **P < 0.005, ***P < 0.001 and ns, not significant. Error bars indicate standard deviation (SD). (B) G1 and G4 mTerc ^−/−^ pup brains (each group from six pooled pup brains) were used to prepare shake‐off glial cultures with 10 parts equivalent DsRed pup brain homogenates (pooled from 12 DsRed pup brains). Repopulation ability of G1 and G4 mTerc ^−/−^ microglia was assessed by quantifying the percentage of the red vs. nonred populations. Flow cytometry controls of nonred (C) and DsRed (D) cells. G4 mTerc ^−/−^ microglia showed a decreased % proportion on four successive shake‐off cultures (H vs. F), while G1 mTerc^−/−^ increased in % proportion with respect to Ds Red (G vs. E) indicating compromised proliferation and repopulation capacity in G4 mTerc ^−/−^ microglia.

Relative telomere length analysis in microglia isolated from young (4 months) and aged (24 months) C57/BL6 inbred mice did not reveal a significant difference in telomere length as determined by quantitative PCR, indicating that microglia do not undergo significant telomere shortening during aging in mice. In contrast to microglia from aged mice (24 months), a significant reduction in telomere length was observed in microglia from 6‐month‐old G3mTerc^−/−^ mice in comparison with age‐matched G1mTerc^−/−^ controls. The telomeres from G3mTerc^−/−^ microglia were significantly shortened compared to aged microglia (Fig. 1A).

To investigate whether telomere shortening affected the proliferative capacity of microglia, we performed a competitive proliferation assay. For this purpose, we prepared postnatal glial cell cultures (P0–P4) from G4mTerc^−/−^, G1mTerc^−/−^, and DsRed pups (expressing red fluorescent protein in all cells). The proliferation of microglia in the postnatal mixed glia cultures also depends on trophic support from the underlying astrocyte layer. To circumvent the possibility of defective astrocyte proliferation hence affecting microglia proliferation, the mixed glia cultures were set up by mixing either G1 mTerc^−/−^ or G4 mTerc^−/−^ with DsRed postnatal brain tissue in a ratio of 1:10. DsRed expression was used to discriminate between microglial cells derived from DsRed and G1 mTerc^−/−^ or G4 mTerc^−/−^ mice.

The relative contribution of red (from DsRed brains) vs. nonred microglia (G1 mTerc^−/−^ or G4 mTerc^−/−^ brains) during proliferation of the postnatal cultures was analyzed using flow cytometry, and these proportions are indicated as percentage populations in flow cytometry graphs from the first to the fourth mitotic shake‐off over a period of 1 month. The results show that while the proportion of G1 mTerc^−/−^ microglia increased from the 1st to the 4th shake‐off (Fig. 1G compared to E), the relative contribution of G4 mTerc^−/−^ microglia decreased during successive mitotic shake‐offs (Fig. 1H compared to F). The cultures generated from G1 mTerc^−/−^ or G4 mTerc^−/−^ brains that were not mixed with DsRed cells showed hardly any red fluorescence (Fig. 1C). Shake‐offs from DsRed cultures were used as a positive control (Fig. 1D). The quantified results of the % proportions are depicted in Fig. 1B. These data show that G4 mTerc^−/−^ microglia have a reduced proliferative capacity compared to G1 mTerc^−/−^ microglia.

### Telomere shortening does not induce many phenotypic and functional alterations in microglia

To determine the activation status of microglia upon telomere shortening, protein expression of several cell surface markers including microglia markers such as CD11b, CD45, F4/80, and CD11c; neuronal communication molecules such as CD200R and CD172a; and antigen presentation molecules such as CD80 and MHC II were analyzed. No difference in expression levels of these proteins between G1 and G3 mTerc^−/−^ mice of 8 months of age was observed (Fig. S1A,B). Transcriptome analysis of pure microglia, directly isolated from 4‐month‐old G1 mTerc^−/−^ and G4 mTerc^−/−^ mice, was performed to determine possible changes in gene expression due to telomere shortening. The gene expression analysis demonstrated only a modest upregulation of *p21* expression in G4 mTerc^−/−^ microglia (Fig. S1C). Interestingly, several genes that have been previously shown to be expressed during microglial activation such as *Egr1, Chi3l3, Chi3l4, Ccl3, Lyz1, Fos, Jun, and Mapk8* were downregulated in G4 mTerc^−/−^ microglia (Fig. S1D).

Expression of phagocytic receptors, such as CD36, CD18, CD204, and CD54 (Fig. S1E) on microglia cell surface, assessed by flow cytometry, was comparable between 8‐month‐old G1 and G3 mTerc^−/−^ mice. To determine whether telomere shortening affected phagocytic capacity, acutely isolated microglia from 8‐month‐old G1 mTerc^−/−^ and G3 mTerc^−/−^ mice were tested in a phagocytosis uptake assay using *E. coli* bacteria coupled to pH Rodo. The pH Rodo dye fluoresces at acidic pH upon fusion of the phagocytic cargo with lysosomes and hence facilitates visualization of only the internalized particles. Analysis of red fluorescence in G1 mTerc^−/−^ and G3 mTerc^−/−^ microglia by flow cytometry showed that the phagocytic intake was similar in both cases (Fig. S1F). Also, DCFDA fluorescence mediated by released of reactive oxygen species (ROS) by microglia was found to be comparable in G1 mTerc^−/−^ and G3 mTerc^−/−^ microglia (8 months) under control conditions and when stimulated with 40 nm ATP (Fig. S1G).

### Increased microglial immune response to endotoxemia in G3 mTerc^−/−^ microglia

**Figure 2:**
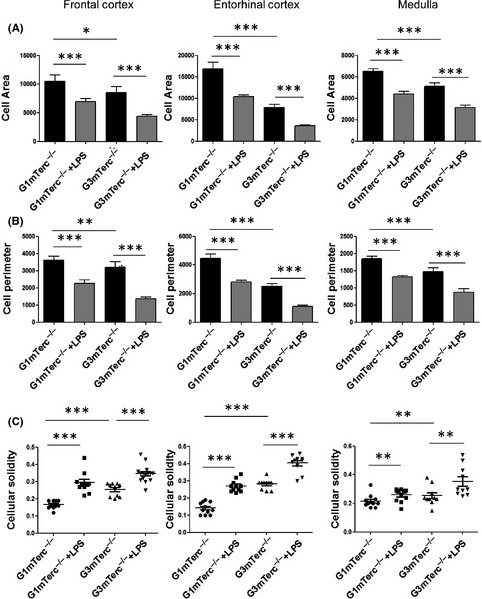
Increased hypertrophy in microglia of late‐generation telomerase knockout mice. Quantification of (A) microglia area, (B) microglia perimeter, and (C) microglia solidity/occupancy. Decrease in cell area, perimeter, and solidity was evident in LPS‐treated G3 mTerc^−/−^ microglia compared to G1 mTerc^−/−^ microglia. At least 10 cells from three animals per group were used for quantification. Asterisks * indicate comparisons for which *P*‐value was significant according to two‐way ANOVA *P < 0.05, **P < 0.005, ***P < 0.001. Error bars indicate standard error of mean (SEM).

Inflammatory reactivity in microglia, based on morphological changes and cytokine expression, was investigated in G1 mTerc^−/−^ and G3 mTerc^−/−^ mice during intraperitoneal injection of lipopolysaccharide. G1 and G3 mTerc^−/−^ mice (10 months) were terminated 24 h after LPS injection for analysis of microglia morphology. There were observable differences in morphology of microglia between G1 mTerc^−/−^ and G3 mTerc^−/−^ brain slices (Fig. S2). A detailed quantitative analysis of microglia morphology was performed in 10‐month‐old G1 mTerc^−/−^ and G3 mTerc^−/−^ brain slices with or without LPS injection. A two‐way ANOVA, to understand the effect of genotype and LPS treatment, showed that there were notable differences in cell area and perimeter between G1 mTerc^−/−^ and G3 mTerc^−/−^ microglia in the brain regions studied including frontal cortex, entorhinal cortex, and medulla. Quantification of the images showed a significant decrease in microglia cell area and perimeter in LPS‐injected G3 and G1 mTerc^−/−^ mice compared to noninjected mice (Fig. 2A,B). Cell solidity (calculated as described in the Materials and methods section) is an effective parameter for identification of subtle changes in microglia morphology at the intermediate stages toward activation (Soltys *et al*., 2001). An increase in cellular occupancy indicates the transition from a ramified state toward hypertrophy. Microglial solidity/occupancy was significantly increased in LPS‐injected G3 and G1 mTerc^−/−^ mice indicating a hypertrophic state (Fig. 2C). Although both genotype and LPS treatment individually were found to be significantly different in most conditions, the combined interaction effect was not significantly different indicating that the extent of morphological changes in LPS‐injected G3mTerc^−/−^ microglia vs. noninjected G3mTerc^−/−^ microglia is comparable with that in G1mTerc^−/−^ microglia.

**Figure 3:**
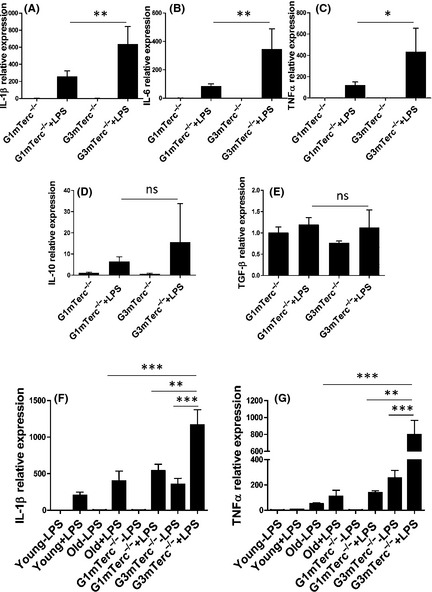
Increased cytokine response of G3 mTerc^−/−^ microglia to LPS challenge. Microglia were sorted as CD11b^high^/CD45^intermediate^ population from G1 (*n* = 4) and G3 mTerc^−/−^ (*n* = 4) brains, after being injected with vehicle (PBS) or with LPS (1 mg kg^−1^, 4 h). Relative mRNA expression of cytokines was quantified by real‐time RT–PCR. The microglia expression of the pro‐inflammatory cytokines IL‐1β (A), IL‐6 (B), and TNF‐α (C) was significantly increased in response to LPS with a much higher expression in G3 mTerc^−/−^ microglia compared to G1 mTerc^−/−^ microglia isolated from 6‐month‐old animals. Anti‐inflammatory IL‐10 (D) TGF‐β (E) expression in response to LPS was not significantly different between G1 and G3 mTerc^−/−^ microglia at this age. Basal expression of cytokines was also negligible at this age. However, at an age of 10 months, G3 mTerc^−/−^ (*n* = 6) microglia show increased secretion of IL‐1β (F) and TNF‐α (G) more than aged microglia isolated from 24‐month‐old mice (C57/Bl6 background; *n* = 4 for young and aged mice). Also, at 10 months, basal expression of these cytokines was higher in G3 mTerc^−/−^ microglia compared to G1 mTerc^−/−^ microglia (*n* = 6). Asterisks * indicate comparisons for which *P*‐value was significant according to one‐way ANOVA *P < 0.05, **P < 0.005, ***P < 0.001 and ns, not significant. Error bars represent SD.

To address microglial inflammatory reactivity, gene expression of pro‐inflammatory cytokines interleukin‐6 (IL‐6), tumor necrosis factor‐alpha (TNF‐α), and interleukin‐1 beta (IL‐1β) and anti‐inflammatory cytokines transforming growth factor‐beta 1 (TGF‐β1) and interleukin 10 (IL‐10) was determined in microglia isolated from 6‐month‐old G1 and G3 mTerc^−/−^ mice, i.p. injected with saline or LPS 3 h prior to isolation. The expression of pro‐inflammatory cytokines was significantly increased in G3 mTerc^−/−^ microglia compared to G1 mTerc^−/−^ microglia in LPS‐injected animals. In saline‐injected G3 mTerc^−/−^ mice, cytokine expression levels were comparable with those of G1 mTerc^−/−^ mice (Fig. 3A–E).

In an independent experiment, the extent of LPS‐induced microglial cytokine expression in microglia from G3 mTerc^−/−^ and physiologically aged mice was compared. LPS was injected i.p. in young (4 months), aged C57/BL6 mice (22 months), G1 mTerc^−/−^ (10 months), and G3 mTerc^−/−^ (10 months) mice. G3 mTerc^−/−^ mice that did not show behavioral alterations such as changes in nesting behavior, severe kyphosis, or hind leg clasping were chosen for this experiment. The results show that the increased pro‐inflammatory cytokine expression in G3 mTerc^−/−^ microglia is much stronger than the expression in aged microglia. Also, at 10 months, G3 mTerc^−/−^ microglia expression of pro‐inflammatory cytokines was detected in the absence of external stimuli (Fig. 3F,G).

### Age‐associated priming genes are not expressed in microglia upon telomere shortening

**Figure 4:**
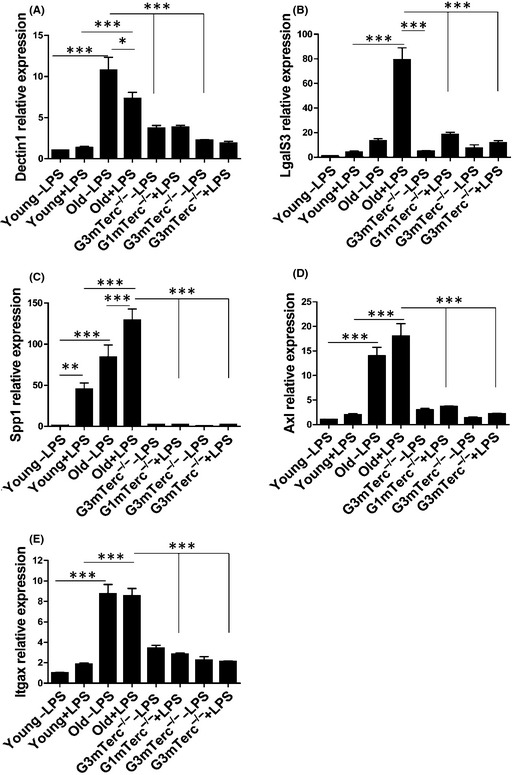
Absence of age‐associated priming genes in G3 mTerc^−/−^ microglia in the presence or absence of LPS. Microglia were sorted as CD11b^high^/CD45^intermediate^ population from G1 and G3 mTerc^−/−^ brains (*n* = 6), after being injected with vehicle (PBS) or with LPS (1 mg kg^−1^, 4 h), and the relative mRNA expression of genes of interest was quantified by real‐time RT–PCR. Age‐associated priming genes (A) Dectin1, (B) LgalS3/Mac2, (C) Spp1/osteopontin, (D) Axl, and (E) Itgax/CD11c were expressed in aged microglia isolated from 24‐month‐old mice (*n* = 4) compared to young 4‐month‐old controls (*n* = 4). However, the expression of these genes was comparable and low between G1 and G3 mTerc^−/−^ microglia in the presence or absence of LPS‐induced inflammation. Asterisks * indicate comparisons for which *P*‐value was significant according to one‐way ANOVA *P < 0.05, **P < 0.005, ***P < 0.001 and ns, not significant. Error bars represent SD.

Age‐related priming in microglia has been previously shown to induce a specific gene expression profile (Hickman *et al*., 2013). We selected genes that were upregulated in ‘primed’ aging microglia and analyzed their expression by quantitative PCR in microglia from young (4 months), aged (24 months), G1, and G3 mTerc^−/−^ mice (10 months). Age‐related priming genes such as *Dectin1*,* LgalS3*,* Spp1*,* Axl,* and *Itgax* were highly expressed in microglia isolated from aged WT mice (Fig. 4A–E). However, microglia isolated from G3 mTerc^−/−^ mice did not show any expression of age‐associated priming genes, independent of i.p. LPS injection.

### Alterations in blood–brain barrier integrity and increased leukocyte infiltration upon telomere shortening in late‐generation mTerc^−/−^ mice

**Figure 5:**
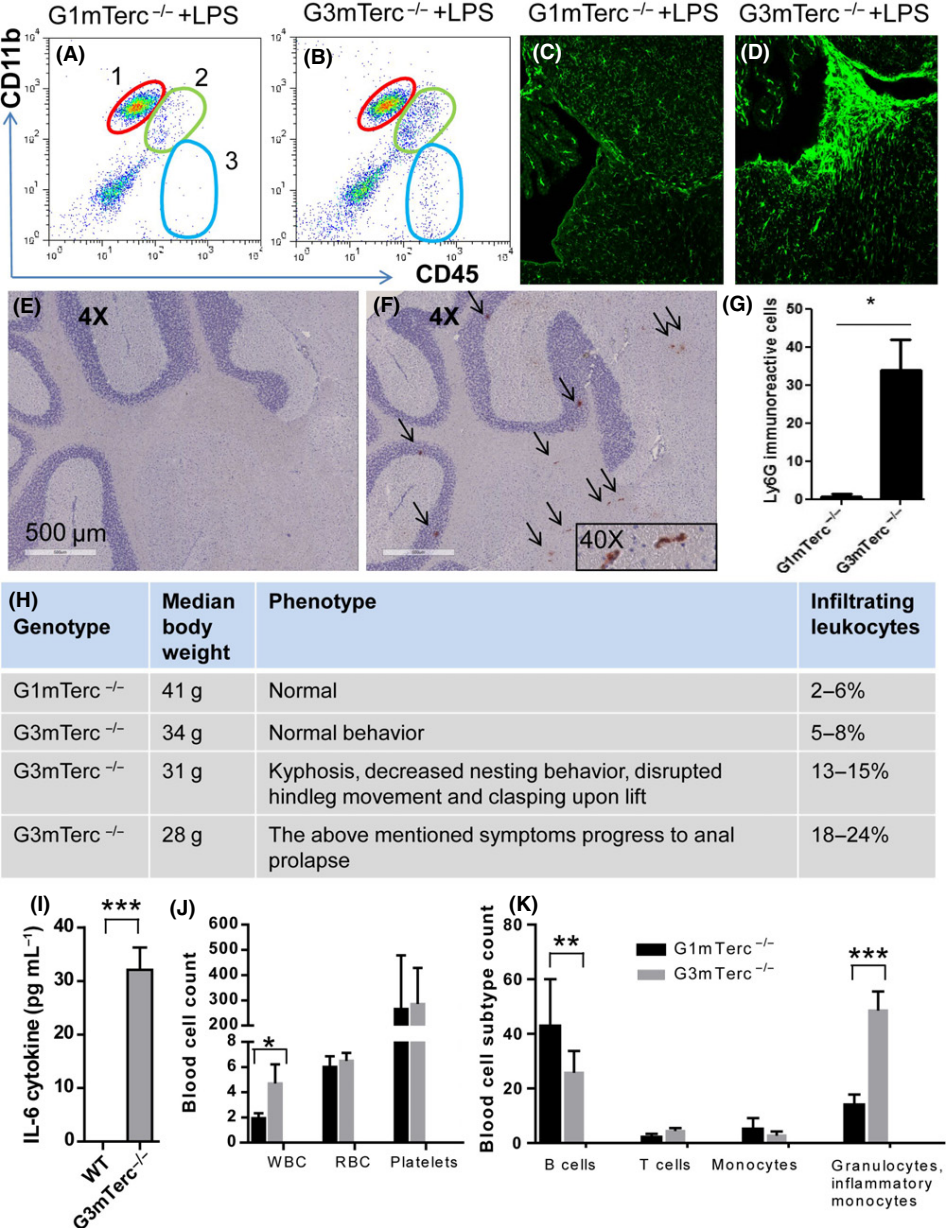
Increased infiltration of blood leukocytes in G3 mTerc^−/−^ brains. CD11b CD45 staining in G1 mTerc^−/−^ (A) vs. G3 mTerc^−/−^ (B) animals. Gate1 indicates microglia, Gate 2 represents infiltrating macrophages, and Gate 3 represents leukocytes. CD11b^low^/CD4^med/high^ infiltrating macrophages and leukocytes were higher in LPS‐injected G3 mTerc^−/−^ animals. CD45‐immunoreactive cells are present around white matter tracts near ventricles in LPS‐injected G3 mTerc^−/−^ mice (D) and not abundant in G1 mTerc^−/−^ (C) brains. Ly6G‐positive neutrophils are higher in G3 mTerc^−/−^ brain parenchyma (F) (*n* = 3) compared to G1 mTerc^−/−^ brains (E; plotted in G) (*n* = 3). Phenotypic presentations in G3 mTerc^−/−^ mice and corresponding CD45^high^ cellular population percentages are tabulated in (H). IL‐6 cytokine levels in G3 vs. G1mTerc ^−/−^ plasma (I). Blood cell counting shows higher number of WBCs in G3 mTerc^−/−^ bone marrow (J). Differential blood cell phenotyping shows higher number of Gr‐1‐positive granulocytes and inflammatory monocytes in circulation compared to B cells and T cells (K). *P < 0.05, **P < 0.005, ***P < 0.001 and ns, not significant. Error bars represent SD.

The BBB strictly regulates communication between the CNS and peripheral circulation. Under conditions of inflammation, circulating leukocytes can pass the BBB and have been shown to contribute to CNS pathologies (Hult *et al*., 2008). Infiltrating leukocytes in the brain are CD45^hi^ (gates 2 and 3) (Kettenmann *et al*., 2011). Flow cytometry analysis of G3 mTerc^−/−^ brains showed increased CD45^hi^ cells upon i.p. LPS injection (gates 2 and 3 in Fig. 5B compared to A). G3 mTerc^−/−^ brains showed a much higher number of CD45‐positive cells particularly near ventricles upon LPS injection than in G1 mTerc^−/−^ brains (Fig. 5C,D). Using a specific marker for neutrophils, Ly6G, infiltrating neutrophils were detected in the G3 mTerc^−/−^ brain parenchyma (Fig. 5E,F). The number of neutrophils in the parenchyma was higher in G3 mTerc^−/−^ compared to G1 mTerc^−/−^ brains (Fig. 5G).

Aging G3 mTerc^−/−^ mice exhibit a range of phenotypic presentations of aging. Even littermates of the same age can range from showing normal behavior to decreased nesting behavior, disrupted hind leg movement with hind leg clasping upon lifting and kyphosis of the spine. Occasionally, these symptoms progress to anal prolapse (upon which the animal is terminated for ethical considerations). The variation in phenotypic presentations might be due to heterogeneity in telomere erosion. However, the progressive worsening of the aging phenotype correlates with the percentage of brain infiltrates. In aged G3 mTerc^−/−^ animals that show degenerative changes such as intestinal dystrophy or anal prolapse, the infiltrating leukocytes constituted up to 24% of the total cell isolate (Fig. 5H). To check whether organ dystrophy causes increased peripheral inflammation, IL6 cytokine measurements in blood samples and analysis of blood cells were performed in bone marrow. IL6 cytokine levels were found to be 30‐fold higher in G3 mTerc^−/−^ plasma samples compared to G1 controls (Fig. 5I). Also, blood cell phenotyping showed higher white blood cells (WBCs) in G3 mTerc^−/−^ blood samples (Fig. 5J). More careful analysis of cellular subtypes showed an increased number of granulocytes and inflammatory monocytes identified as SSC^high^ CD11b+ Gr‐1+ cells in circulation in G3 mTerc^−/−^ (SSC indicates side scatter in flow cytometry; Fig. 5K) indicating increased inflammation. B cells (CD45R+ B220+) were decreased in G3mTerc^−/−^ animals. T cells (CD3+) and monocytes (SSC^med^ CD11b+) were not significantly different (Fig. 5K).

**Figure 6:**
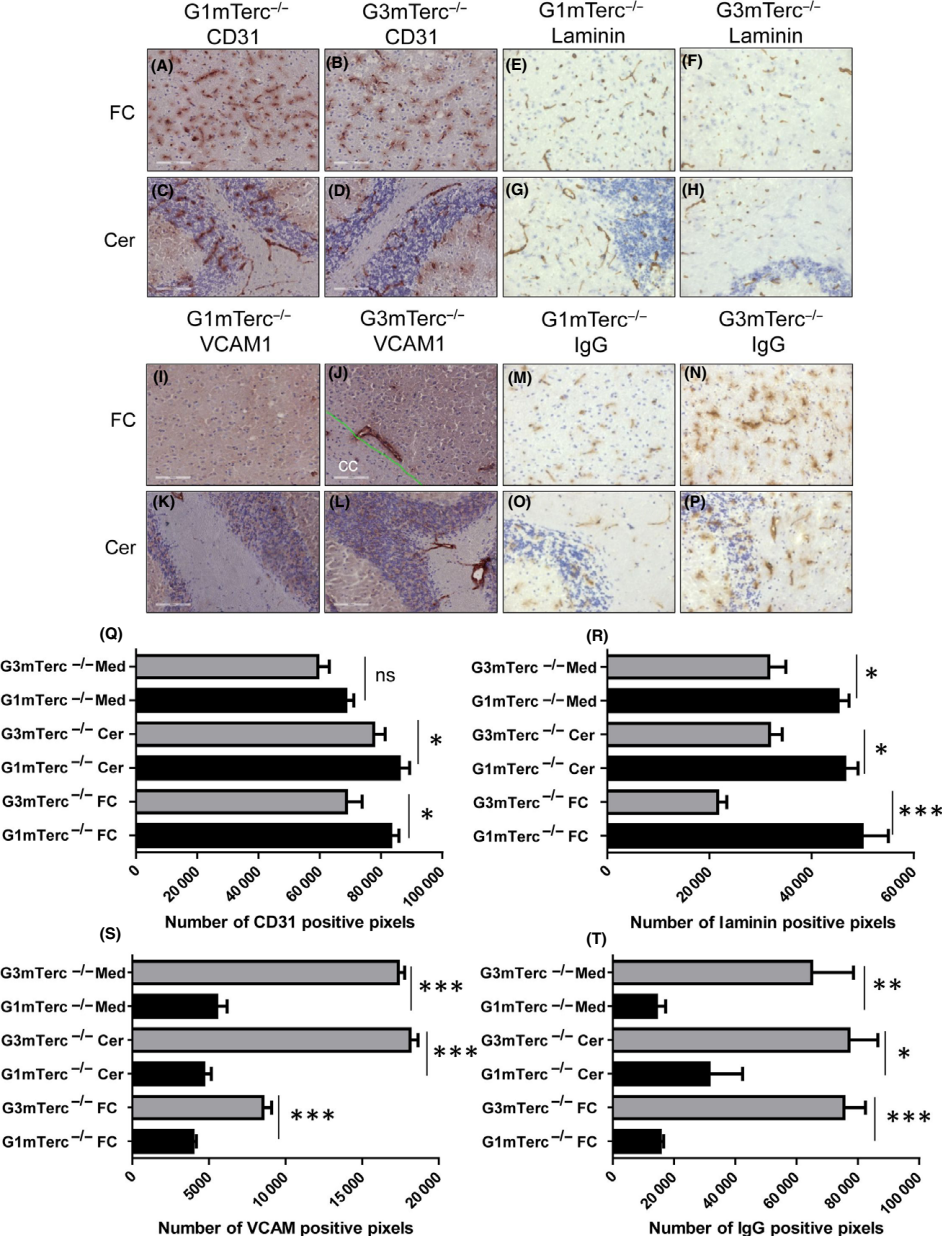
Compromised blood–brain barrier and endothelial activation in G3 mTerc^−/−^ brains. CD31 shows slightly decreased vessel density in G3 mTerc^−/−^ brains (*n* = 3; B, D) compared to G1 mTerc^−/−^ counterparts (*n* = 3; A, C). CD31 immunostaining quantified as positive pixels between G1 and G3 mTerc^−/−^ brains (Q) Laminin shows decreased and stunted vessels in G3 mTerc^−/−^ brains (*n* = 3; F, H) compared to G1 mTerc^−/−^ counterparts (*n* = 3; E, G). Laminin immunostaining quantified as positive pixels between G1 and G3 mTerc^−/−^ brains (R). VCAM, a marker of endothelial activation, was upregulated in G3 mTerc^−/−^ brains (*n* = 3; J, L) compared to G1 mTerc^−/−^ brains (*n* = 3; I, K). Quantification of VCAM‐positive pixels (S) in different brains regions. IgG permeability was assessed by immunoreactivity to IgG. IgG1‐immunoreactive serrated, leaky blood vessels were abundant in G3 mTerc^−/−^ brains (*n* = 3; N, P) compared to G1 mTerc^−/−^ brains (*n* = 3; M, O). Quantification of IgG‐positive pixels (T) in different brains regions. FC denotes frontal cortex, Cer denotes cerebellum, and Med denotes medulla. Asterisks * indicate comparisons for which *P*‐value was significant according to one‐way ANOVA *P < 0.05, **P < 0.005, ***P < 0.001 and ns, not significant. Error bars indicate SD.

Increased infiltration is an indication of decreased BBB integrity. Telomerase ablation has previously been shown to affect neuroinflammation and the BBB in an experimental mouse model of stroke (Zhang *et al*., 2010). We checked the status of the BBB by immunohistochemical evaluation of blood vessel appearance and density in 10‐month‐old G1 and G3 mTerc^−/−^ brains for CD31 antigen, which stains endothelial cells, and laminin, which stains the basement membrane in the vessel walls. In G3 mTerc^−/−^ mice, CD31 immunoreactivity was slightly decreased in the frontal cortex (FC) (Fig. 6A,B) and cerebellum (Cer) (Fig. 6C,D) compared to G1 mTerc^−/−^ mice (Fig. 6Q). Laminin immunoreactivity on the other hand was noticeably decreased in G3 mTerc^−/−^ brains (Fig. 6R), and this decrease was observed in all brain regions studied: FC, Cer, and medulla (Med) (Fig. 6E–H), while the decrease in laminin staining was most pronounced in the FC. Endothelial activation in blood vessels upregulates proteins including VCAM1, which play a role in leukocyte adhesion and infiltration. VCAM1 expression is increased in G3 mTerc^−/−^ brains (Fig. 6I–L) and particularly predominant in deep white matter regions. A hallmark of BBB alteration is the presence of IgG in the brain parenchyma. Circulating IgG cannot normally enter the brain parenchyma across an intact BBB (Bullard *et al*., 1984; Seitz *et al*., 1985). IgG1 permeability assessed by immunohistochemistry showed intact blood vessels in G1 mTerc^−/−^ brain tissue, whereas G3 mTerc^−/−^ brains show large, serrated IgG1 immunoreactive vessels. Together, these data indicate alterations in blood vessels and a decrease in BBB integrity in G3 mTerc^−/−^ mouse brains.

## Discussion

Differences in telomere biology between mice and humans have been acknowledged previously (Calado and Dumitriu, 2013). Based on telomere length, the use of mouse models for studying physiological aging particularly in regard to the role of telomeres in aging is a matter of debate (Wright & Shay, 2000; Gomes *et al*., 2011). Age‐related telomere shortening and its consequences cannot be studied effectively in normal aging mice. Telomere length analysis in the aged human brain showed no detectable correlation with age in some studies (Allsopp *et al*., 1995), while in others, there was an observed correlation (Nakamura *et al*., 2007; Lukens *et al*., 2009) which was speculated to be due to change in telomere lengths of a subset of brain cells (Lukens *et al*., 2009). In accordance, a study analyzing telomere lengths in pure human microglia isolated from the brain shows significant shortening and predicted propensity for dementia (Flanary *et al*., 2007). Here, we show that microglia in G3 mTerc^−/−^ mice display considerable telomere shortening compared to G1 mTerc^−/−^ mice, analogous to results in aged human microglia (Flanary *et al*., 2007). On the other hand, microglia isolated from young (3 months) and aged (24 months) mice under healthy conditions do not show significant telomere shortening. Therefore, the G3 mTerc^−/−^ mouse model seems more suitable than wild‐type aging mice to study the effects of telomere shortening on microglia.

Telomere shortening in mTerc^−/−^ mice was apparent only after several generations of inbreeding, as telomeres shorten at a rate of 4.8 ± 2.4 kb per *Terc* ^*−/−*^ generation (Blasco *et al*., 1997). Consequently, the third generation of mTerc^−/−^ mice (G3 mTerc^−/−^) showed premature aging phenotypes hallmarked by gray hair, humped back, compromised fertility, and decreased life span (Herrera *et al*., 1999). In line with this, telomere shortening in late‐generation mTerc^−/−^ mice compromised the proliferative potential of cultured microglia. It is, however, not possible to exclude that differential survival ability of microglia from late‐generation mTerc^−/−^ mice could in effect produce a similar experimental outcome. Microglial telomere shortening, as observed in G3 mTerc^−/−^ mice (at 6 months of age), does not affect marker expression or vital microglia functions such as phagocytosis and free radical production (analyzed at 8 of months age). In spite of their reduced proliferative potential upon extended culturing, gene expression profiling from G4 mTerc^−/−^ microglia showed only modest changes in gene expression levels compared to G1 mTerc^−/−^ microglia. Interestingly, G4 mTerc^−/−^ microglia show an increase in p21 mRNA. p21 is a p53 target gene, implicated in cell cycle arrest, tumor suppression and used as a marker of cellular senescence (Serrano *et al*., 1997). Interestingly, genes previously reported in activated microglia such as *Egr‐1, CCl3, Chi3l3, Chi3l4, and Lyz1* (McMahon & Monroe, 1996; Chang *et al*., 2001; Babcock *et al*., 2003; Friedle *et al*., 2011) and in proliferation such as *Fos, Jun,* and *Mapk8* (Yamasaki *et al*., 2014) were found to be mildly but significantly downregulated in G4 mTerc^−/−^ microglia.

A study investigating the role of telomere shortening on plaque pathology using APP23 mice (a mouse model for Alzheimer's disease) crossed with telomerase knockout mice (mTerc^−/−^) indicated that telomere shortening improved the spatial learning ability and decreased plaque load (Rolyan *et al*., 2011). The study showed a change in morphology of G3 mTerc^−/−^ microglia but did not display differences in expression of microglia proteins such as MHC II that are altered during activation. Our detailed quantification of morphology showed that microglia in G3 mTerc^−/−^ brain showed differences in cell area, perimeter, and solidity compared to G1 mTerc^−/−^ microglia, but no differences in surface protein expression or functionality were noticed. We reasoned that telomere shortening could alter immune response in the presence of an external stimulus without changing the basal cellular profile drastically. Previously, aged microglia have been shown to display enhanced pro‐inflammatory activity in response to LPS (Sierra *et al*., 2007). I.p. injection of LPS was chosen as the external stimulus. Enhanced cytokine expression was found in G3 mTerc^−/−^ microglia. Notably, 10‐month‐old G3 mTerc^−/−^ microglia showed an enhanced pro‐inflammatory response higher than 24‐month‐old wild‐type microglia. Although the extent of changes in morphology is similar in G1 and G3 mTerc^−/−^ microglia in the presence of LPS, the functionality in terms of cytokine response was found to be distinctly different. These data emphasize the limited use of morphological parameters to assess microglia functionality.

Enhanced and persistent neuroimmune activity of microglia associated with aging is referred to as microglia priming (Perry & Holmes, 2014) and shows a unique gene expression signature (Hickman *et al*., 2013). The gene expression signature of aging microglia was found to be highly similar to the profile observed in a mouse model of progressive DNA damage accumulation as a consequence of ERCC1 dysfunction, the Ercc1 mutant mice (Ercc1^Δ/−^) (Holtman *et al*., 2015). In this mouse model, the change in microglia phenotype to a primed state was shown to be the response to neuronal genotoxic stress (Raj *et al*., 2014). Interestingly, both telomere dysfunction and DNA damage accumulation have been shown to signal via a common p21‐mediated DNA damage response mechanism in neurons (Jurk *et al*., 2012; Sperka *et al*., 2013). Analysis of genes associated with age‐associated microglia priming showed that microglia isolated from aged mice highly expressed these genes, but G3 mTerc^−/−^ microglia do not display any expression of priming genes in the presence or absence of peripheral inflammation, indicating that telomere shortening does not result in microglia priming. Telomerase deficiency has been shown to induce DNA damage response and inflammation in late‐generation postmitotic mTerc^−/−^ neurons (Jurk *et al*., 2012). Therefore, the absence of microglia priming in this mouse model might even seem surprising. This might be due to several factors. Telomere dysfunction and DNA damage accumulation likely induce varied DNA lesions, and despite shared downstream mechanisms (Jurk *et al*., 2012; Sperka *et al*., 2013), it is unknown whether all downstream signaling due to these triggers is the same. Clearly, some but not all stressors that cause genomic instability impact priming in microglia. Also, in addition to genomic DNA damage accumulation, ERCC1 dysfunction also results in transcriptional initiation defects which neurons in particular could be vulnerable to Kamileri *et al*. (2012).

An impaired BBB could offer an explanation for the increased response to LPS by mTerc^−/−^ microglia. In an experimental model of stroke, telomerase deficiency, induced by knocking out the protein component of telomerase enzyme (TERT) results in a defective BBB (Zhang *et al*., 2010). This mouse model likely reflects the effect telomere deficiency on BBB rather than that of telomere shortening. In the G3 mTerc^−/−^ brains, which suffer from telomere shortening during aging, a decrease in blood vessel laminin, the main component of the basement membrane, and a slight decrease in CD31 protein expressed by endothelial cells were noticed. Furthermore, increased IgG1 permeability in G3 mTerc^−/−^ brains is indicative of a deficient BBB function. Taken together, these results show that the BBB is compromised in G3 mTerc^−/−^ mice. This could be the consequence of endothelial vascular senescence due to telomere shortening, previously proposed as a mechanism of age‐associated endothelial dysfunction (Erusalimsky, 2009). Indeed, aging has also been shown to result in telomere shortening in the endothelium (Aviv *et al*., 2001). In general, brain aging and AD have been shown to be accompanied by a decrease in microvessel density and cerebral blood flow (Brown & Thore, 2011). However, the extent of decrease in laminin is notably more pronounced than that of CD31. In addition to endothelial cells, specific subtypes of laminin are also produced by astrocytes and pericytes (Yao *et al*., 2014). Previously, it has been shown that dysregulation in production of astrocytic laminin can compromise BBB by affecting pericyte differentiation and vascular smooth muscle cell function (Chen *et al*., 2013; Yao *et al*., 2014). Recently, by employing a high‐resolution MRI method, blood to brain transfer of gadolinium bound to plasma proteins (shows entry of large molecules that cannot usually enter the brain) was measured regionally in a quantitative manner. Montagne *et al*. (2015) showed BBB disruption in the aging human hippocampus and cellular injury to pericytes. It cannot be ruled out that pericytes, astrocytes, or vascular smooth muscle cell dysfunction due to telomere shortening affects BBB integrity in this mouse model.

In addition, increased infiltrating leukocytes that enter the CNS due to a compromised BBB can also amplify a central immune response in conditions of peripheral inflammation via brain microglia. This process is controlled by cytokine and chemokine signaling and has been shown to affect brain microglia under conditions of inflammation remote to the CNS such as hepatic inflammation (Riazi *et al*., 2008; D'Mello *et al*., 2009). G3 mTerc^−/−^ mice after 8 months of age start showing prominent signs of intestinal dystrophy and in extreme cases resulting in anal prolapse. In aged G3 mTerc^−/−^ mice (10 months), indications of peripheral inflammation were detected: increased numbers of circulating granulocytes and inflammatory monocytes and high cytokines. Mice with intestinal dystrophy have increased numbers of infiltrating leukocytes in the brain indicating that the increased peripheral inflammation together with changes in BBB facilitates infiltration of immune cells in brain parenchyma. Together, these factors alter the inflammatory behavior of microglia upon telomere shortening and explain the increased basal expression of cytokines in G3 mTerc^−/−^ microglia at 10 months of age compared to 6 months. We have shown that, at resting conditions, microglia in G3 mTerc^−/−^ mice do not show a dystrophic or primed phenotype due to intrinsic telomere shortening. However, after i.p. injection of LPS, an enhanced pro‐inflammatory response of these microglia is observed, which most likely is the consequence of a compromised BBB and may be amplified by the presence of infiltrated immune cells in the brain parenchyma.

## Funding

Part of the work has been performed at the UMCG microscopy and imaging center (UMIC), which is sponsored by NWO‐grants 40‐00506‐98‐9021 and 175‐010‐2009‐023. Help with morphometry imaging by Klaas Sjollema is acknowledged. Operators of central FACS facility of UMCG, Geert Mesander, Henk Moes, and Roelof Jan van der Lei, are acknowledged for their support. The research leading to these results has received funding from the European Community's Seventh Framework Programme (FP7/2007–2013) under grant agreement No. HEALTH‐F2‐2010‐ 259893. KB was supported by the German Research Council (DFG): FOR1336, grant numbers: DFG BI 668/5‐1; BI668/2‐2. This work was also supported by the Mouse Clinic for Cancer and Aging through a RoadMap grant from the Netherlands Organization for Scientific Research (NWO). The authors greatly acknowledge Dr. Pim van der Harst, Dr. Herman Silljé, Dr. Liza S. M. Wong, and Inge Vreeswijk‐Baudoin of the Dept of Cardiology, UMCG, for help with the mTerc mouse colony. B6.Cg‐Tg(CAG‐DsRed* MST)1Nagy/J (dsRed) transgenic mice was a kind gift from Dr. Gerald de Haan's laboratory.

## Conflict of interest

No conflicts of interest.

## Supporting information


**Fig. S1** Telomere shortening results in modest phenotypic, gene expression or functional changes in microglia.

**Fig. S2** Morphological hypertrophic response to LPS in microglia in the late generation telomerase knockout mice.

**Data S1** Materials and methods.
**Table S1** Antibody information for immunophenotyping.
**Table S2** PCR primer information.
