# Supplementary figures and images for: Enhanced microglial pro‐inflammatory response to lipopolysaccharide correlates with brain infiltration and blood–brain barrier dysregulation in a mouse model of telomere shortening
Source: Aging Cell. 2015 Aug 3;14(6):1003–13. doi: 10.1111/acel.12370 (PMC4693462; doi:10.1111/acel.12370)

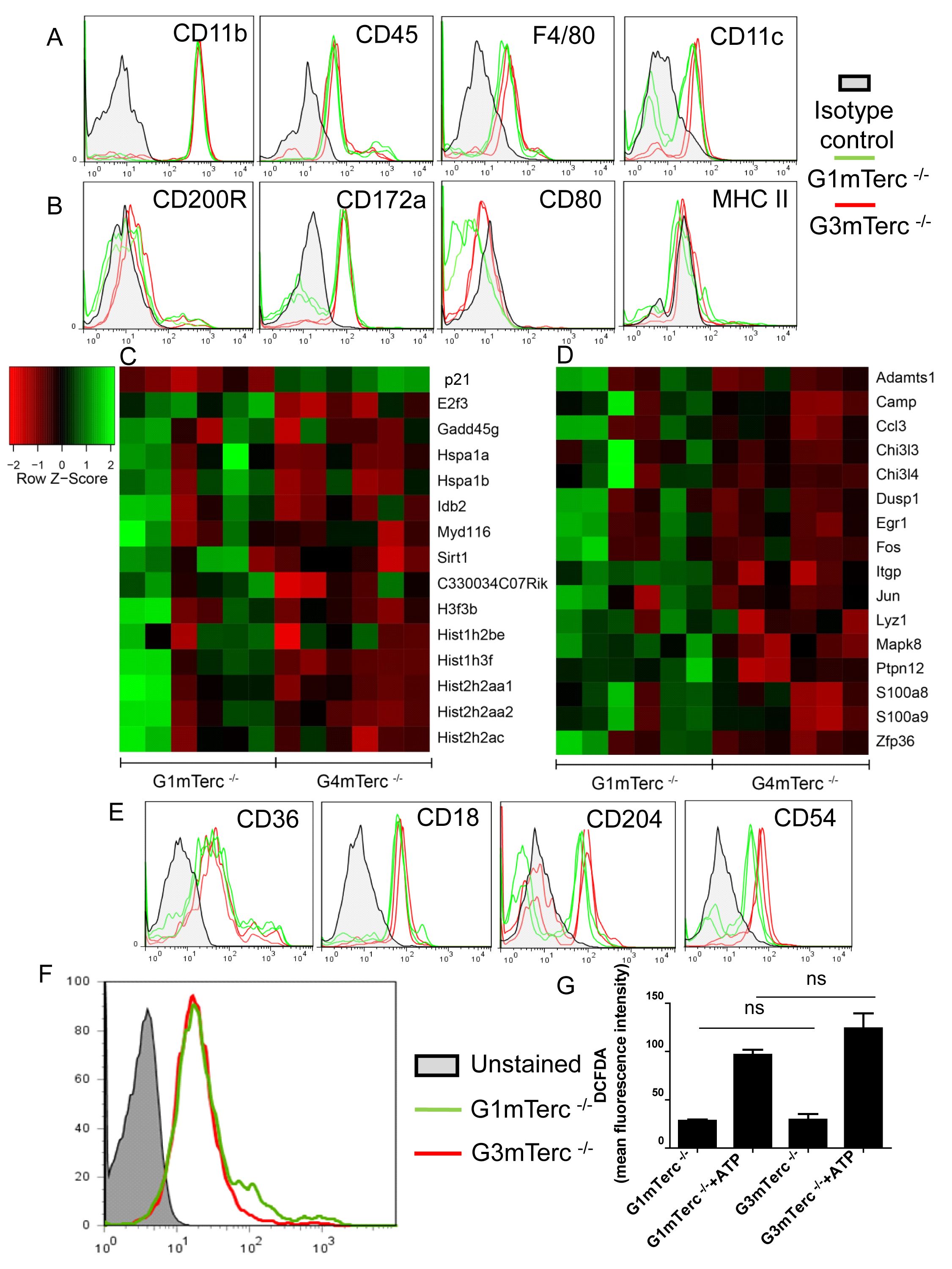

Supplement: Supplementary file 1 — Fig. S1 Telomere shortening results in modest phenotypic, gene expression or functional changes in microglia. [file ACEL-14-1003-s001.jpg]

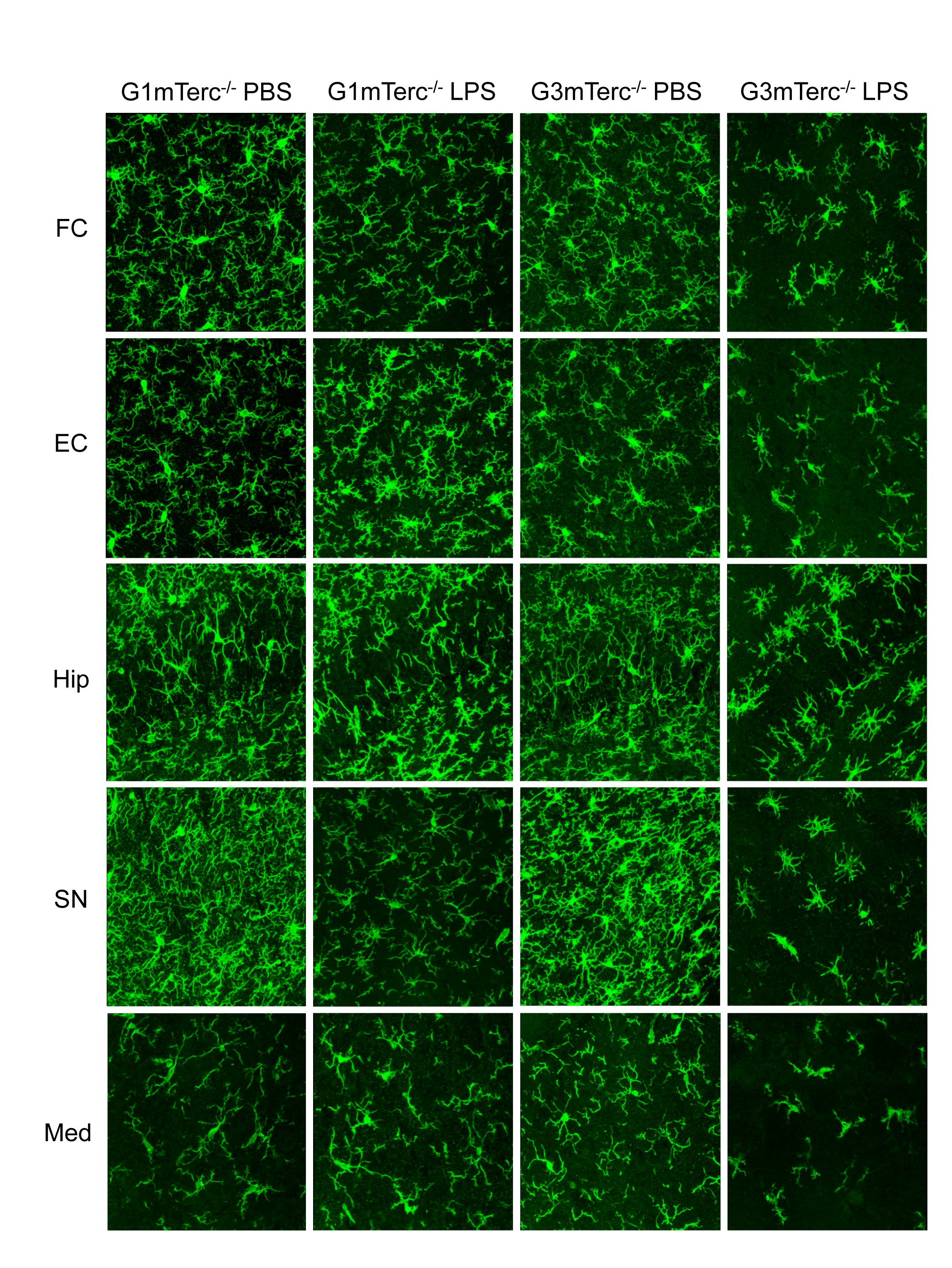

Supplement: Supplementary file 2 — Fig. S2 Morphological hypertrophic response to LPS in microglia in the late generation telomerase knockout mice. [file ACEL-14-1003-s002.tif]
