# Supplementary material for: Enhanced microglial pro‐inflammatory response to lipopolysaccharide correlates with brain infiltration and blood–brain barrier dysregulation in a mouse model of telomere shortening
Source: Aging Cell. 2015 Aug 3;14(6):1003–13. doi: 10.1111/acel.12370 (PMC4693462; doi:10.1111/acel.12370)
Supplement: Supplementary file 3 — Data S1 Materials and methods. Table S1 Antibody information for immunophenotyping. Table S2 PCR primer information. [file ACEL-14-1003-s003.docx]

**Materials and methods for supplementary data:**

**Competitive proliferation assay**

Glial cell culture from postnatal brain tissue allows microglia to be “shaken-off” at high purity from astrocyte beds as they proliferate once a week for about four rounds of division (Biber et al., 2001). Briefly, after removal of the meninges and brainstem, brains were triturated in medium A (HBSS with 0.6% glucose, 15 mM HEPES buffer, and 1% penicillin/streptomycin) containing 0.25% trypsin and 1% DNase I (Gibco, Grand Island, NY) to obtain a single cell suspension. The cell pellet from G1 mTerc^-/-^ ( isolated from postnatal day 4, P4) or G4 mTerc^-/-^ (P1) was counted and mixed with 10 times equivalent cell numbers from DsRed brains (P0). The DsRed protein is expressed under the actin promoter in all cells of these mice. Mixing with DsRed cells was done for the following reason: The microglia in glia cultures require astrocyte support to proliferate and survive. It was unclear if astrocyte proliferation is also affected in mTerc^-/-^ mice. In order to ensure sufficient trophic support from astrocytes in both cultures and to be able to distinguish cells added for trophic support, the glia cultures were set up by mixing G1 mTerc^-/-^ or G4 mTerc^-/-^ cells with DsRed pup-homogenates. The mixed pellet was then plated with culture medium containing DMEM with 4.5 g/l glucose, 2 mM Glutamine, 1% penicillin/streptomycin and 1% Sodium pyruvate, supplemented with 10% FCS in 75cm^2^ flasks in a 1:1 mix with conditioned medium of the same composition from exogenous wild type mixed glia cultures. Cultures were maintained for 3 weeks in 5% CO_2_ at 37ºC. After 3 weeks, microglial cells were harvested by orbital shaking which separates them from the underlying astrocyte layer and the repopulation efficiency of the cells were analyzed by flow cytometry analysis.

**RNA amplification and microarray analysis**

Microglia were sorted as CD11b^high^/CD45^intermediate^ /DAPI^neg^ population in RNA lysis buffer and RNA was extracted as mentioned above. Concentration of RNA was measured on a nanodrop (ND1000) and RNA integrity checked using bioanalyzer. Additional RNA amplification was performed with Nugen Ovation RNA amplification Kit. Subsequently, RNA was labeled and hybridized onto the Illumina Mouse WG6 Genomestudio (version 1.9.0) was used to generate expression values. Raw data was preprocessed and analysised by project R (version 2.13.1) and BioConductor package Limma (version 1.8.18) (Smyth., 2005). Background correction was done with inferred negative probes, and subsequently, quantile normalization and log2 transformation was applied. Not expressed probes were filtered out below detection level of p<0.05 in all samples. Differential gene expression was done using the rank product test (Hong., 2011) with a percentage of false predictions cut-off of 0.1. Heatmaps were generated using heatmap.2 function of Bioconductor Package gplots.

**Phagocytosis assay**

After isolation from the Percoll gradient (75-25%) interface, cells were counted and resuspended in 1 ml culture medium and seeded in 6 well plates as 100,000 cells/well. 1.5 hours after seeding, medium was replaced with culture medium containing 25 μg/ml of **pHrodo™ E.coli BioParticles® conjugate** (Invitrogen). After 6 hours, the fluorescence emission was recorded in FACS Calibur flow cytometer, BD (emission maxima of pH Rodo is 585nm) and the flow cytometric measurements were analysed using FlowJo software and plotted as histograms.

**Determination of reactive oxygen species production**

Production of reactive oxygen species by acutely isolated human microglia was determined by detecting the conversion of H_2_DCFDA (2’,7’-dichlorodihydrofluorescein diacetate) into fluorescent DCF (2',7’-dichlorofluorescein) with flow cytometry. 100 μl of cell suspension containing 2×10^4^ cells was incubated with and without H_2_DCFDA. 5 μM of H_2_DCFDA was added to the cells and incubated for 20 minutes in the presence or absence of adenosine-5′-triphosphate (ATP, 40 nM), after which fluorescence was measured using flow cytometry. The mean fluorescence intensity of DCF after subtraction of the background fluorescent values from cells only was plotted.

**Immunohistochemistry**

Immunohistochemical staining using 4% paraformaldehyde in 0.1M phosphate buffer (pH 7.4) overnight at 4 °C and cryoprotected with 25% sucrose and cut in to 50 μm thick sections. Parallel free-floating sections were incubated for endogenous peroxidase blocking (2% H_2_O_2_ in 70% methanol for 10 min), followed by 1 h in Blocking Buffer solution (BB) containing 5% normal goat serum or fetal calf serum in phosphate buffered saline (PBS) containing 0,1% Triton X-100 (Fluka) (PBST) for 30 minutes at room temperature. Afterwards, sections were incubated 48 hours in PBST at 4 °C with anti-Iba1 (Wako, Japan), anti-Mac2 (Cedarlane, Canada), anti-laminin (MP Biomedicals, USA), Anti-IgG (Vector Laboratories Inc, USA). Negative controls devoid of primary antibody were also included. After washes with PBS, sections were incubated at room temperature for 1 h with either biotinylated anti-rabbit (1:200; BA-1000) secondary antibody (from Vector Laboratories Inc, Burlingame, CA). Sections were then rinsed in PBS and incubated with horseradish peroxidase streptavidin (1:400; SA-5004; Vector Laboratories Inc, Burlingame, CA, USA). The peroxidase reaction was visualized by incubating the sections in Tris buffer containing 0.5 mg/ml 3, 3′- diaminobenzidine (DAB) and 0.33 μl/ml H_2_O_2_. Sections were then dehydrated and coverslipped with DePeX.

Alternatively, for some primary antibodies such as Ly6G (Clone 7/4, Biosource International), CD45, CD31 (BD pharmingen, San Diego, USA), VCAM1 (Clone M/K-1.9, ATCC, Manussas VA, USA) snap frozen brains (16 µm) were used for immunostaining. Briefly, slices were fixed with acetone for 10 min, air-dried and were incubated with primary antibody for 2 h at room temperature followed by washing in PBS. Primary rat anti mouse antibodies were diluted in 5% fetal calf serum (FCS) in PBS. Endogenous peroxidase was blocked by incubation with peroxidase blocker from the DAKO envision kit (Cambridgeshire, UK) for 5 min. Detection was performed with rabbit anti-rat secondary antibodies (preabsorbed, Vector Laboratories, Burlingame, CA, USA) diluted to 1:300 in PBS/ 5% FCS, which was followed by anti-rabbit HRP incubation (DAKO envision kit). Between antibody incubations, slides were extensively washed with PBS. Peroxidase activity was detected with 3-amino-9-ethylcarbazole (DAKO envision kit), and sections were counterstained with Mayer’s haematoxylin (Merck, Darmstadt, Germany).

Slides were scanned using a ScanScope XT Digital Slide Scanner (Aperio) at a resolution of 0.25 mm/pixel (100,000 pix/in) and data was analyzed with the positive pixel count algorithm (Imagescope). These algorithms make use of a color deconvolution method (Ruifrok and Johnston, 2001) to separate stains, so that quantification of individual stains avoided cross contamination. A minimum of 5 fields per brain region (20x magnification) were quantified for each animal. The data is representative of 3 independent animals from each cohort and is depicted as total number of positive pixels ± SD.

**Quantitative morphological analysis of microglia**

Images of brain sections with Iba1 staining performed on free-floating brain sections (at every 0.5 µm depth throughout 45µm thickness) were acquired as Z-stacks to analyze the whole cell architecture. Maximum projection images were examined by creating a region of interest (ROI) manually that would include the cell of interest entirely. All analyses were performed using ImageJ. The length of the external and internal borders were measured independently and the results were summed up to measure the perimeter of the cell, Pc. Solidity was calculated as S = Ac/Ah, where Ac is the area of cell, Ah is the convex hull area. The convex hull is the smallest convex polygon circumscribed on the cell silhouette. The figure for convex hull was drawn manually using the polygon selection tool from the ImageJ program. All parameters were calculated from thresholded 8-bit images. In all cases, the number of microglial cells analyzed was 30-40 per group (*n* = 3 mice; atleast 10 cells per region per animal).

**Telomere length measurements**

Genomic mouse DNA isolated from pure microglial cell populations (AllPrep DNA/RNA Micro Kit (Qiagen) according the manufacturer’s protocol) was used for the measurement of the average telomere length using quantitative PCR. Primers (telomere and 36B4), primer sequences, primer concentrations and thermal cycling profiles for both primers were as described by Callicott and Womack (2006) in their mouse telomere measurement protocol. The PCR was run on a 7900HT Taqman (Applied Biosystems) in a reaction volume of 11 µl and the input amount of the experimental samples was 12.5 ng of genomic DNA. The ratio of telomere (T): 36B4 (S) was calculated and the average of these ratios was reported as the average telomere length ratio (ALTR). T/S >1 and T/S <1 indicates average telomere length greater and less than standard DNA. To serve as a reference for standard curve calculation, a mix of 3x G1 mTerc ^-/-^ and G3 mTerc^-/-^  or a mix of 3x old and young microglia  DNA samples having the highest concentration was prepared and serially diluted over a 30-fold range from 2 to 60 ng per well for both telomere and 36B4 primers similar to the procedure followed in Callicott and Womack (2006).

**Endotoxemia model**

Animals were intraperitoneally injected with 1 mg/kg lipopolysaccharide (LPS) (*E.coli* 0111:B4; Sigma-Aldrich, St. Louis, MO, L4391) or saline. 4h (for RNA isolation) or 18h (for immunohistochemistry) after injection, the animals were sacrificed and the brains isolated and processed for appropriate analysis.

**Enzyme-linked immunosorbent assay (ELISA)**

Il-6 levels were measured in blood plasma using commercially available ELISA kits according to manufacturer’s instructions (Biolegend).

**Differential blood cell counting**

Bone marrow cells were isolated from both femora. Single cell suspensions were obtained by flushing the femur and the total number of nucleated cells was assessed using a Coulter Counter (Coulter Electronics, Dunstable, England) followed by a standard NH_4_Cl erythrocyte lysis. Subsequently, cells were incubated with 5% normal rat serum for 15 minutes at 4°C, after which cells were stained with biotinylated lineage-specific antibodies : anti-CD45R, anti-B220, anti-Gr-1, anti-CD3, anti-CD11b, (all antibodies from Pharmingen, San Diego, CA; http://www. bdbiosciences.com) for 40 minutes at 4°C. Cells were subsequently incubated with Streptavidin-PE (Pharmingen) for 40 minutes at 4°C and strained through a 35-μm cell strainer (Becton, Dickinson, Bedford, MA; http://www.bd.com), after which cells were quantified using a MoFlo flow cytometer (DakoCytomation, Fort Collins, CO; http://www.dakocytomation.com).

**Supplementary figure legends:**

**Supp. Fig. 1: Telomere shortening results in modest phenotypic, gene expression or functional changes in microglia.**

No changes in protein expression for microglia activation markers were noticed in G1 and G3 mTerc ^-/-^ (n=6) microglia in (A) microglia markers (B) neuronal interaction molecules and antigen presentation molecules. Changes in gene expression were restricted to less than 50 genes between G1 and G4 mTerc ^-/-^ microglia (n=6). Upregulation is indicated by green and downregulation is indicated by red. (C, D) Upregulated gene, p21 and downregulated genes in G4 mTerc ^-/-^ microglia are represented in the heatmap. (E) Expression of phagocytic receptors such as CD36 , CD18 , CD204 , CD54 on microglia cell surface as assessed by flow cytometry were comparable between 8 months old G1 (green histograms) and G3 mTerc^-/-^ (red histograms) mice (n=6). (F) Ex-vivo isolated microglia from G1 and G3 mTerc ^-/-^ mice did not show any compromise in phagocytic uptake of pH Rodo coupled *E.coli* bacteria (n=6). (G) ROS production as measured by DCFDA fluorescence using flow cytometry showed no differences between G1 and G3 mTerc ^-/-^ microglia (n=6).

**Supp. Fig. 2: Morphological hypertrophic response to LPS in microglia in the late generation telomerase knockout mice.**

Z-Stack confocal images of microglia stained for Iba1 in G1 and G3 mTerc ^-/-^ (10 months old; n=3) injected with PBS or LPS (1mg/Kg weight of the animal) 24 hours prior to termination. Regions depicted include Frontal cortex (FC), Entorhinal cortex (EC), Hippocampus (Hip), Substantia Nigra (SN), Medulla (Med). G1 and G3 mTerc ^-/-^ show differences in morphology in in all brain regions.. LPS injected G1 and G3 mTerc ^-/-^ brain slices are proportionally different from non-injected controls.

**Supplementary table 1: Antibody information for immunophenotyping**

| Antibody | Catalog number | Company | Flurochorme |
| --- | --- | --- | --- |
| CD11b | 12-0112 | ebiosciences | Phycoerythrin |
| CD45 | 11-0451 | ebiosciences | Fluorescein isothiocyanate |
| CD80 | 11-0801 | ebiosciences | Fluorescein isothiocyanate |
| MHC class II | 11-5321 | ebiosciences | Fluorescein isothiocyanate |
| CD11c | 11-0114-81 | ebiosciences | Fluorescein isothiocyanate |
| F4/80 | 11-4801-81 | ebiosciences | Fluorescein isothiocyanate |
| CD200R | 123909 | BioLegend | Fluorescein isothiocyanate |
| CD172a | 560107 | BD Biosciences Pharmingen | Phycoerythrin |
| CD36 | 12-0361-81 | ebiosciences | Phycoerythrin |
| CD18 | 11-0181-81 | ebiosciences | Fluorescein isothiocyanate |
| CD54 | 11-0541-81 | ebiosciences | Fluorescein isothiocyanate |
| CD204 | MCA1322A488 | ADb Serotec | Alexafluor 488 |

**Supplementary table 2: PCR primer information**

| Gene | Accession number | Forward sequence | Reverse sequence | Amplicon |
| --- | --- | --- | --- | --- |
| IL-1β | NM-008361 | ATCTTTGAAGAAGAGCCCATCC | TGTAGTGCAGTTGTCTAATGGG | 91 bp |
| IL-6 | NM-031168 | CCTCTCTGCAAGAGACTTCCATCCA | GGCCGTGGTTGTCACCAGCA | 64 bp |
| IL-10 | NM_010548 | AAGGGTTACTTGGGTTGCCA | TTTCTGGGCCATGCTTCTCT | 91 bp |
| TGFβ | NM-011577 | GAACCAAGGAGACGGAATACAG | GGAGTTTGTTATCTTTGCTGTCAC | 74 bp |
| TNFα | NM-013693 | TCTTCTGTCTACTGAACTTCGG | AAGATGATCTGAGTGTGAGGG | 111 bp |
| Dectin1 | NM-020008 | CCCAACTCGTTTCAAGTCAG | AGACCTCTGATCCATGAATCC | 81 bp |
| Spp1 | NM-009263 | AGCAAGAAACTCTTCCAAGCA | CATCCGAGTCCACAGAATCC | 117 bp |
| LgalS3 | NM-010705 | CAGGATTGTTCTAGATTTCAGGAG | TGTTGTTCTCATTGAAGCGG | 73 bp |
| Axl | NM-001190974 | TGAAGCCACCTTGAACAGTC | GCCAAATTCTCCTTCTCCCA | 117 bp |
| Itgax | NM-021334 | CTGGATAGCCTTTCTTCTGCTG | GCACACTGTGTCCGAACTCA | 113 bp |
